# Cleavage of the APE1 N-Terminal Domain in Acute Myeloid Leukemia Cells Is Associated with Proteasomal Activity

**DOI:** 10.3390/biom10040531

**Published:** 2020-03-31

**Authors:** Lisa Lirussi, Giulia Antoniali, Pasqualina Liana Scognamiglio, Daniela Marasco, Emiliano Dalla, Chiara D’Ambrosio, Simona Arena, Andrea Scaloni, Gianluca Tell

**Affiliations:** 1 Department of Medicine, University of Udine, 33100 Udine, Italy; lisa.lirussi@medisin.uio.no (L.L.); giulia.antoniali@uniud.it (G.A.); emiliano.dalla@uniud.it (E.D.); 2 Department of Pharmacy, University of Naples “Federico II”, 80134 Naples, Italy; liana.sco@gmail.com (P.L.S.); daniela.marasco@unina.it (D.M.); 3 Proteomics & Mass Spectrometry Laboratory, ISPAAM, National Research Council, 80147 Naples, Italy; chiara.dambrosio@cnr.it (C.D.); simona.arena@cnr.it (S.A.); andrea.scaloni@cnr.it (A.S.)

**Keywords:** apurinic apyrimidinic endonuclease/redox effector factor 1, cytoplasmic nucleophosmin 1, proteasome, acute myeloid leukemia, proteolysis

## Abstract

Apurinic/apyrimidinic endonuclease 1 (APE1), the main mammalian AP-endonuclease for the resolution of DNA damages through the base excision repair (BER) pathway, acts as a multifunctional protein in different key cellular processes. The signals to ensure temporo-spatial regulation of APE1 towards a specific function are still a matter of debate. Several studies have suggested that post-translational modifications (PTMs) act as dynamic molecular mechanisms for controlling APE1 functionality. Interestingly, the N-terminal region of APE1 is a disordered portion functioning as an interface for protein binding, as an acceptor site for PTMs and as a target of proteolytic cleavage. We previously demonstrated a cytoplasmic accumulation of truncated APE1 in acute myeloid leukemia (AML) cells in association with a mutated form of nucleophosmin having aberrant cytoplasmic localization (NPM1c+). Here, we mapped the proteolytic sites of APE1 in AML cells at Lys31 and Lys32 and showed that substitution of Lys27, 31, 32 and 35 with alanine impairs proteolysis. We found that the loss of the APE1 N-terminal domain in AML cells is dependent on the proteasome, but not on granzyme A/K as described previously. The present work identified the proteasome as a contributing machinery involved in APE1 cleavage in AML cells, suggesting that acetylation can modulate this process.

## 1. Introduction

Apurinic/apyrimidinic endonuclease 1 (APE1) is a multifunctional and essential protein protecting cells from oxidative and alkylative insults [1], which maintains the genome stability through a DNA repair action. It is also able to modulate gene expression through redox signaling and transcriptional activities. Regardless of transcriptional control, modulation of the APE1 interactome under different conditions and post-translational modifications (PTMs) at specific residues can modify the chemical characteristics and the structural conformation of the protein, redirecting APE1 toward a specific function. The disordered N-terminal region (35 amino acids) plays a key role in: (i) regulating APE1 subcellular localization [2]; (ii) mediating APE1 binding to RNA and to most of the other protein interactors [3,4]; (iii) controlling the catalytic activity on abasic DNA [5]; (iv) acting as an acceptor of different PTMs, such as acetylation [5,6,7], ubiquitination [8,9], and proteolysis [6,10,11,12,13,14]. The proteolytic cleavage of the N-terminal portion of APE1 is an irreversible modification that has not been well elucidated in its molecular/functional characteristics. Although the loss of the N-terminus of APE1 does not alter the enzyme endonuclease activity, it affects its subcellular distribution as a consequence of the removal of the nuclear localization signal of the protein [5]. The formation of the above-mentioned truncated protein was reported to be dependent on Granzyme activity [11,12]. Clear evidence has suggested that the truncated form is not retained within nucleoli but displays a cytoplasmic rather than a nuclear distribution [11,12]; on the other hand, conflicting data were reported on the effect of proteolytic cleavage on APE1 endonuclease and redox activities [11].

Recently, we demonstrated that the expression of the NPM1c+ mutant protein in the myeloid cell line OCI/AML3 and in blasts from acute myeloid leukemia (AML) patients is accompanied by APE1 relocalization to the cytoplasm [13]. Interestingly, we observed an accumulation of truncated APE1 in the cytoplasmic compartment, suggesting that this protein form is destabilized in cells expressing NPM1c+ protein [13]. The proteolytic site on APE1 in AML cells, as well as the protease responsible for the generation of its truncated form, are still unknown.

In this study, we performed *in vitro* proteolysis experiments using different recombinant APE1 protein forms as substrates. The obtained results clearly demonstrated that: (i) the proteolytic events in AML cells involve the removal of the protein N-terminus, as we previously demonstrated in other cells [13], and specifically cleavage at Lys31 and Lys32; (ii) proteolysis is not ascribable to a granzyme-like activity, contrarily to previous investigations [11,12]; (iii) the cleavage of the APE1 N-terminal domain in AML cells depends on the proteasome complex activity; (iv) possibly, acetylation can modulate this process.

## 2. Materials and Methods

### 2.1. Cell Culture and Materials

OCI/AML2 and 3 cells were grown in alpha-MEM (Euroclone, Milan, Italy) supplemented with 20% fetal bovine serum, 100 U/mL penicillin and 10 µg/mL streptomycin sulfate. Jurkat and HL-60 cells were grown in RPMI-1640 medium (Euroclone, Milan, Italy); all cells were supplemented with 10% fetal bovine serum (FBS, EuroClone, Milan, Italy), L-glutamine (2 mM) (Euroclone, Milan, Italy), penicillin (100 U/mL) and streptomycin (100 mg/mL) (Euroclone, Milan, Italy), and were cultured in a humidified incubator, at 37 °C, in a 5% *v*/*v* CO_2_ atmosphere. All chemical reagents used for treatments were supplied from Sigma-Aldrich (St. Louis, MO, USA) unless otherwise specified.

### 2.2. Preparation of the Cell Extracts and Anti-FLAG Co-Immunoprecipitation

Preparation of total cell lysates, cytoplasmic and nuclear extract were performed as previously described [3]. HeLa cells were transiently transfected with FLAG-tagged APE1 mutants (APE1^WT^, APE1^31-34Ala^, APE1^K4pleA^, APE1^NΔ33^) using the Lipofectamine 2000 Reagent (Invitrogen, Carlsbad, CA, USA), according to the manufacturer’s instructions, and collected 24 h after transfection. Co-immunoprecipitation experiments were performed as already described [15].

### 2.3. In Vitro Proteolysis

Whole, cytoplasmic or nuclear OCI/AML2-3, Jurkat or HL-60 cell lysates were incubated with FLAG-tagged APE1 mutants in Granzyme cleavage buffer (50 mM Tris-HCl, pH 7.5, 1 mM CaCl_2_ and 1 mM MgCl_2_) in a final volume of 20 μL, for the indicated times, at 37 °C, as previously reported [11]. Reactions were blocked with 4X Laemmli sample buffer and corresponding products were resolved by SDS-PAGE; resulting gels were immunoblotted for FLAG (F1804, Sigma-Aldrich, St. Louis, MO, USA) or stained with Coomassie R250 (Sigma-Aldrich, St. Louis, MO, USA).

### 2.4. Western Blotting Analysis

For Western blotting analysis, the indicated amounts of cell extracts were resolved in 12% SDS-PAGE and transferred onto nitrocellulose membranes (Sigma-Aldrich, St. Louis, MO, USA). Normalization was performed by using a monoclonal anti-tubulin antibody (T0198, Sigma-Aldrich, St. Louis, MO, USA), a polyclonal anti-actin (A2066, Sigma-Aldrich, St. Louis, MO, USA) or a monoclonal anti-FLAG (F1804, Sigma-Aldrich, St. Louis, MO, USA). All the other antibodies used were from Novus Biologicals (Abingdon, UK). Blot images were acquired and analyzed by using an Odissey CLx Infrared Imaging system (LI-COR GmbH, Bad Homburg, Germany).

### 2.5. Peptide Synthesis

A peptide substrate was designed as corresponding to the portion (26-36) present within the APE1 N-terminal region, having a FRET couple at N-[DABCYL, 4-(4-dimethylaminophenylazo) benzoic acid] and C-[EDANS, 5-(2-aminoethyl) aminonaphthalene-1-sulfonic acid] terminal ends. The peptide was synthesized and purified as already reported [16]. The peptide identity and purity (>95%) was ascertained by an LC-ESI-IT-MS analysis that was carried out with a Surveyor HPLC system (Thermo Fisher Scientific, Waltham, MA, USA) containing a photo diode array (PDA), which was connected on-line with a LCQ DECA XP mass spectrometer (Thermo Fisher Scientific, Waltham, MA, USA) equipped with an OPTON ion source, operating at 4.2 kV needle voltage and 320 °C. Peptide analysis was carried out with a narrow-bore C18 BioBasic column (50 × 2 mm). The purified peptides were lyophilized and stored at −20 °C until their use. Peptide stock solutions were prepared at 2 mM and stored frozen until their use.

### 2.6. Proteolytic Assay

Cell lysate samples (3 µg) were pre-incubated in 50 mM Tris-HCl, pH 7.4, for 5 min at 37 °C; they were added with the above-mentioned peptide substrate, which was present at a concentration 1–100 μM. Final peptide concentration values were estimated through absorbance measurements of the corresponding DABCYL-group (extinction coefficient at 478 nm: 32000 M^−1^ × cm^−1^). After addition of the lysate at 37 °C, peptide enzymatic hydrolysis was measured by recording corresponding fluorescence emission at 460 nm after excitation at 330 nm for 200 min. All experiments were performed in 384 low-volume-well plates (well volume 20 µL) with an OPTI PLATETM instrument (PerkinElmer, Waltham, MA, USA). Experiments were run in triplicate, and corresponding results were reported as average values ± standard deviation (SD).

To determine the cleavage site(s) in the peptide, a reaction mixture containing 100 μM substrate solved in the above-described buffer was added with the cell lysate (3 μg), at 37 °C for 1 h; the corresponding reaction products were analyzed by LC-ESI-IT-MS, as reported above.

### 2.7. RNA Isolation, cDNA Synthesis and PCR

The total RNA from cell lines was extracted with the SV Total RNA isolation System Kit (Promega, Madison, WI, USA). One microgram of total RNA was reverse transcribed using the iScript cDNA synthesis Kit (Bio-Rad, Hercules, CA, USA), according to the manufacturer’s instructions. A semi-quantitative RT-PCR was performed with RedTaq Polymerase (Sigma-Aldrich, St. Louis, MO, USA). Primers used for the detection of GZMA and GZMB were previously described [17].

### 2.8. Recombinant Protein

The expression and purification of recombinant proteins from *E. coli* was performed as previously described [5,15]. When recombinant proteins were used for *in vitro* assays, the acronym rAPE1 was reported in the text.

### 2.9. Proteomic Analyses

#### 2.9.1. Purification of Proteases

Cytoplasmic extracts from serum-starved (60 min) OCI/AML3 cells (about 4 mg) were loaded on a HiTrap SP column (GE Healthcare, Chicago, IL, USA). Then, the resulting flow-through was directly loaded on a HiTrap Benzamidine column (GE Healthcare, Chicago, IL, USA), which was eluted with 50 mM glycine, pH 3. Experiments were carried out on an AKTA Prime FPLC system (GE Healthcare, Chicago, IL, USA). Resulting elution fractions were tested with the above-mentioned *in vitro* proteolysis assay, resolved in SDS-PAGE, and stained with Coomassie R250 (Sigma-Aldrich, St. Louis, MO, USA). Positive fractions were then pooled, and subjected to proteomic analysis for protease identification.

#### 2.9.2. Protein Identification

The above-mentioned elution fractions were resolved by 12% SDS-PAGE, and the resulting whole gel lanes were cut into ten portions. An analogous treatment was performed for a mock sample. The gel slices were triturated, *in-gel* reduced, S-alkylated with iodoacetamide, and trypsinolyzed [18]. The gel particles were extracted with 25 mM ammonium bicarbonate/acetonitrile (1:1 *v*/*v*), and recovered peptides were desalted on μZip-TipC18 (Millipore, Burlington, MA, USA) devices, using 50% *v*/*v* acetonitrile, 5% *v*/*v* formic acid as an eluent. Peptide mixtures were analyzed by nanoLC-ESI-LIT-MS/MS with a LTQ XL mass spectrometer (Thermo Fisher Scientific, San Jose, CA, USA) equipped with a Proxeon nanospray source connected to an Easy-nanoLC (Proxeon, Odense, Denmark) [19]. The peptides were resolved on an Easy C18 column (10 × 0.075 mm, 3 μm) (Proxeon, Odense, Denmark). The mobile phases were 0.1% *v*/*v* aqueous formic acid and 0.1% *v*/*v* formic acid in acetonitrile, running at 300 nL/min. The gradient parameters have previously been published [7]. The spectra were acquired in the range *m*/*z* 400–2000; the acquisition parameters have previously been reported [7]. The raw data files from nanoLC-ESI-LIT-MS/MS experiments were searched by the MASCOT search engine (version 2.2, Matrix Science, London, UK) against a non-redundant UniProtKB database containing *H. sapiens* protein sequences (2018, 174238). The database searching parameters have already been described [7]. The candidate proteins with at least two assigned peptides with an individual confidence level of 95% were considered as properly identified. The definitive peptide assignment was always associated with manual spectra visualization and verification.

#### 2.9.3. Functional Enrichment Analysis

The identified proteins from proteomic analysis were functionally characterized using the Cytoscape plugin ClueGO querying the KEGG metabolic database (10/12/2019) [20,21]. A two-sided hypergeometric test (corrected using the Benjamini-Hochberg method to control the false discovery rate) was applied to find enriched and depleted terms (adjusted *p* ≤ 0.05). The related terms sharing similar associated genes were fused to reduce redundancy (minimal number of genes = 4; minimal percentage = 4.0).

### 2.10. In Vitro Cleavage Assay with Purified Proteasome

The cleavage reaction assay was performed using 125 ng of purified recombinant proteins and 500 ng of 20S proteasome (Enzo Life Sciences, Farmingdale, NY, USA) in 50 mM Tris-HCl pH 7.5, 25 mM KCl, 10 mM NaCl, 1 mM MgCl_2_ and 1 mM DTT, at 37 °C (final reaction volume 20 μL). After 1 h of incubation, the reaction was blocked with 4× Laemmli sample buffer. The reaction products were then resolved by SDS-PAGE, and immunoblotted for APE1.

## 3. Results

### 3.1. APE1 Is Cleaved at the N-Terminal Region in OCI/AML3 Cells

**Figure 1:**
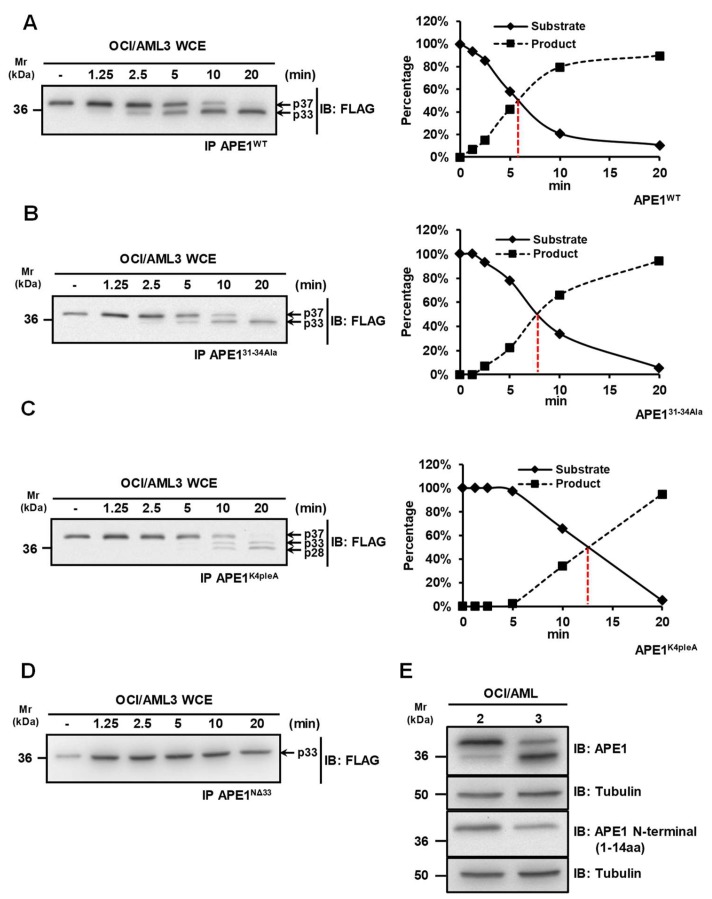
A protease activity is responsible for the cleavage of the APE1 N-terminal domain. (**A**–**D**) Kinetic measurements for the protease activity towards different APE1 mutants. Whole OCI/AML3 cell extracts (10 µg) were incubated for the indicated times, at 37 °C, with immunopurified FLAG-tagged APE1 recombinant proteins expressed in HeLa cells in the wild-type form (APE1^WT^) (**A**), the APE1^31-34Ala^ form where residues 31-34 were mutated to Ala (**B**), the APE1^K4pleA^ form where Lys27, Lys31, Lys32 and Lys35 were mutated to Ala (**C**), and the APE1^NΔ33^ form lacking 33 amino acids at protein N-terminus (**D**). Reactions were separated onto 12% SDS-PAGE, immunoblotted and analyzed using an antibody specific for the FLAG-tag. A representative Western blotting analysis shows the cleavage of APE1 at different times. In the diagrams on the right, the quantification of the densitometric signals of the non-cleaved form of APE1 (S) and the truncated form (P) is reported. (**E**) The N-terminal domain of APE1 is truncated in OCI/AML3 cells. Representative Western blotting analysis on OCI/AML 2 and 3 whole cell extracts (10 µg), showing the N-terminal truncation of APE1 in OCI/AML3 cells. Antibodies used are indicated on the right-hand side. Tubulin was used as loading control.

Previous studies showed that APE1 is targeted by granzymes A and K, which are able to generate truncated protein products (APE1^NΔ31-35^) that are not capable of repairing damaged DNA, thus leading to cellular apoptosis [11,12,22]. We previously reported that a truncated APE1 form can be detected in OCI-AML3 cells expressing the NPM1c+ mutant [13]. In order to map the cleavage site in APE1 and to identify the protease(s) responsible for protein cleavage, we performed *in vitro* proteolysis experiments using whole cells extracts of OCI-AML3 cells that were incubated with purified FLAG-tagged APE1 recombinant proteins from *ad hoc* engineered HeLa cells [7,23]. For these assays, we used different recombinant APE1 protein forms as substrates: (i) wild-type APE1 (APE1^WT^); (ii) an APE1 mutant in which residues Lys31, Lys32, Asn33 and Asp34 were mutated to Ala (APE1^31-34Ala^); (iii) an APE1 mutant, in which residues Lys27, Lys31, Lys32 and Lys35 were mutated to Ala (APE1^K4pleA^), mimicking constitutive acetylation; (iv) an APE1 truncated form lacking the N-terminal portion of 33 amino acids (APE1^NΔ33^) (Figure 1). While the proteolysis kinetics of the wild-type protein was very quick, showing a 50% of cleavage upon 6 min of incubation (Figure lA), amino acid substitutions in mutated variants significantly interfered with the proteolytic event (Figure 1B–D). The 31-34Ala substitution slowed-down the kinetics of about 3 min (Figure 1B), while the K4pleA one lowered the kinetics down to 13 min of incubation, also causing a change in the pattern of proteolysis (Figure 1C). Notably, APE1^NΔ33^ was completely resistant to proteolysis in the investigated time range (Figure 1D), confirming that the proteolysis involved the N-terminal region of APE1.

Additional experiments demonstrated that the above-mentioned proteolytic events involve the removal of the N-terminal portion of APE1. Indeed, when whole OCI/AML extracts were analyzed by Western blotting, full length APE1 was recognized by both an anti-APE1 antibody raised against the C-terminus part of the protein and an antibody recognizing the first 14 amino acids of APE1 N-terminus. Conversely, the truncated form was recognized by the first antibody but not by the latter (Figure 1E). Together with those reported in the previous paragraph on APE1^WT^, APE1^31-34Ala^, APE1^K4pleA^ and APE1^NΔ33^proteolysis patterns and kinetics, these data clearly demonstrated that the observed APE1 degradation involved the removal of the N-terminal protein portion, possibly targeting proteolysis in the segment 31–34.

### 3.2. Characterization of the Proteolytic Sites for APE1 Cleavage

In order to unveil the cleavage site(s) within APE1 N-terminal region and to obtain enzymatic parameters for this proteolytic reaction, an internally quenched peptide spanning protein residues 26-36 (DABCYL-^26^SKTAAKKNDK^36^E-EDANS), including all acetylable Lys residues (Lys27, Lys31, Lys32 and Lys35), was synthesized and used as substrate for the APE1-directed protease present in the OCI-AML3 cell extract.

The hydrolytic events toward the substrate were monitored by real-time measuring of changes in fluorescence. In this case, a potent enhancement of signal upon cleavage is achieved as result of the presence of the donor/acceptor pair constituted by EDANS and DABCYL groups [16]. Time course experiments were performed with different substrate concentrations (1–100 µM) at various enzyme/substrate ratios, and the reaction was monitored for at least 200 min (Figure S1).

**Figure 2:**
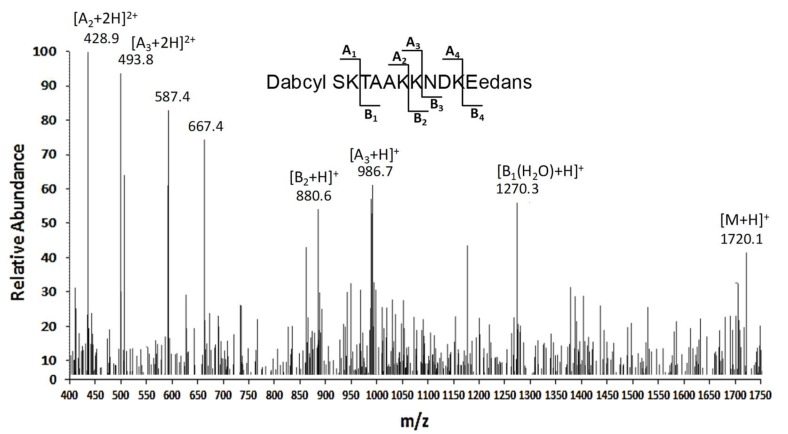
Characterization of the proteolytic sites in a peptide substrate containing the APE1 26-36 portion. ESI-IT-MS analysis of the digest from the treatment of DABCYL-^26^SKTAAKKNDK^36^E-EDANS with a whole OCI/AML3 cell extract. The peaks corresponding to the substrate and hydrolysis products are indicated (*m*/*z*, mass-to-charge ratio).

The digestion mixtures were then directly analyzed through LC-ESI-IT-MS experiments (Figure 2), and resulting signals allowed for the deduction of information on the corresponding reaction products, and consequently on the hydrolysis sites. In particular, the peptide fragment DABCYL-^26^SKTAA^31^K was primarily detected in this mixture, as deduced by the presence of the signal related to (A_2_ + 2H)^2+^ ion at *m/z* 428.9; consistently, the peak at *m/z* 880.6 corresponding to MH^+^ ion of the complementary peptide ^32^KNDKE^36^-EDANS (B_2_ + H)^+^ was also identified. A consistent proteolytic cleavage at Lys32 was also observed, based on the identification of the peptide Dabcyl-^26^SKTAAKK^32^ showing (A_3_ + H)^+^ and (A_3_ + 2H)^2+^ ions at *m/z* 986.7 and 493.8, respectively. In addition, the signal at *m/z* 1270, which was associated with the adduct (M + H_2_O − H)^+^ ion of the fragment TAAKKNDK^36^E-EDANS, also suggested Lys27 as minor potential peptide cleavage site.

Altogether, these data clearly suggested Lys31 and Lys32 as the major cleavage sites in APE1 N-terminal region.

### 3.3. Granzymes Are not Involved in APE1 Proteolysis Observed in OCI/AML3 Cells

**Figure 3:**
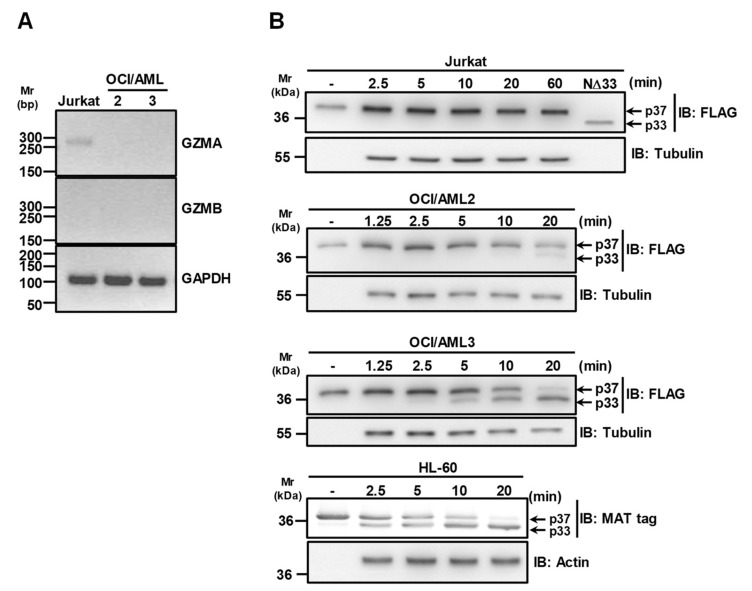
Granzymes A and B are not responsible for APE1 cleavage in OCI/AML cells. (**A**) Granzymes A and B are not expressed in OCI/AML cells. Granzyme A (GZMA) and B (GZMB) expression in OCI/AML2 and OCI/AML3 cell lines were assayed by PCR. Jurkat cDNA was used as positive control. GAPDH expression was used as housekeeping gene. (**B**) APE1 is cleaved in OCI/AML3 but not in the Jurkat cell line expressing GZMA. Representative Western blotting analysis on *in vitro* proteolysis assays carried out using whole cell extracts from OCI/AML2, OCI/AML3, Jurkat and HL-60 cell lines. Antibodies specific for the FLAG-tag or MAT-tag were used. Tubulin and actin were used as loading controls.

Since Granzyme B (GZMB) and A (GZMA) have been reported as able to cleave the N-terminal region of APE1 [11], RT-PCR analysis on RNA extracted from OCI/AML2 and OCI/AML3 cells was carried out to check the expression of these proteases in the aforementioned cells. The data reported in Figure 3A clearly show that neither OCI/AML2 nor OCI/AML3 cells express this form of GZMA and GZMB, in contrast to Jurkat cells used as a positive control for GZMA expression [24]. In addition, the reported cleavage of APE1^31-34Ala^ and APE1^K4pleA^ (Figure 1B,C) definitively excluded a major contribution by GZMA, as these mutants are resistant to GZMA degradation [11].

To discriminate between OCI/AML cells and to evaluate if the absence of truncated cytoplasmic APE1 in extracts of OCI/AML2 cells [13] was due to a differential extent of protease activity in this line, with respect to OCI/AML3, we performed and compared proteolysis experiments with cells extracts from OCI/AML2 and OCI/AML3 cell lines. We also used Jurkat cell extracts as positive control for GZMA expression (Figure 3B). The obtained data clearly indicated that the proteolytic activity on APE1 was significantly reduced in OCI/AML2 cells, compared to the OCI/AML3 counterparts. In addition, the absence of any truncated APE1 form using Jurkat cell extracts further supported the notion that this proteolytic activity is not possibly related to GZMA-mediated degradation. To further support the evidence for a GZMA-independent mechanism of proteolysis of APE1, we also used the HL-60 cell line that does not express this protease [17]. The data reported in Figure 3B clearly show that, despite GZMA absence, the proteolytic activity observed on APE1 was apparent, confirming previous data [22,25]. Altogether, these data supported a model in which NPM1c+ expression is associated with APE1 N-terminal cleavage in a GZMA-independent manner, which is concurrent to its cytoplasmic accumulation.

### 3.4. The Proteasome Machinery Is Involved in APE1 Cleavage

**Figure 4:**
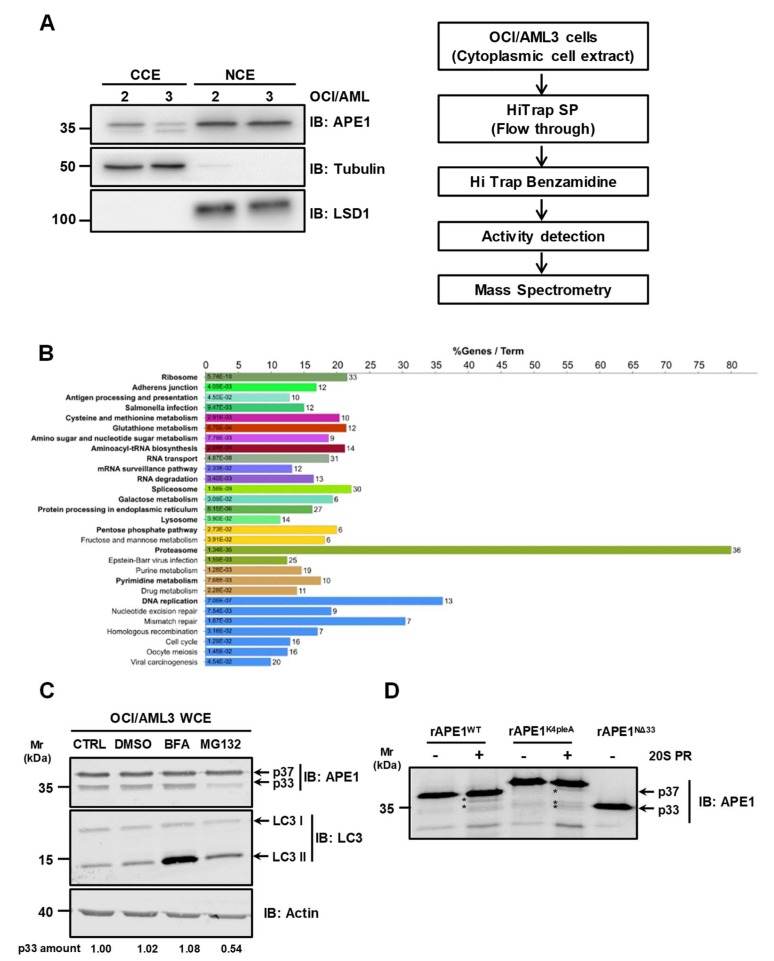
The 20S proteasome is involved in APE1 cleavage. (**A**) Purification strategy used for the isolation of the protease(s) responsible for APE1 truncation in OCI/AML3 cells. Schematic depiction of the workflow used in this study. A OCI/AML3 cytoplasmic lysate (≈ 10^8^ cell equivalents) was loaded onto a HiTrap SP Sepharose column. The resulting flow-through, enriched for negative charged proteins, was loaded on a HiTrap Benzamidine column; serine-proteases retained were then eluted and each fraction was screened for protease activity. Positive fractions were subjected to proteomic analysis for protein identification. Representative Western blotting analysis on nuclear and cytoplasmic extracts of OCI/AML 2 and 3 cell lines, which shows the cytoplasmic localization of the truncated APE1 form in OCI/AML3 cells. Tubulin and LSD1 were used as markers of cytoplasmic and nuclear localization, respectively. (**B**) Functional annotation of the proteins identified in the activity-based positive fractions as deriving from proteomic analysis. Functionally enriched terms (Benjamini-Hochberg adjusted *p* ≤ 0.05) were identified using the Cytoscape plugin ClueGO, querying the KEGG pathway metabolic database; results were summarized as bar charts. Bars length represents the percentage of overall proteins associated with the functional term, which are also present in the examined positive fractions; the exact number of protein associated with each term is shown on the right of each bar, while the Benjamini-Hochberg adjusted *p*-value is shown inside the bars. Each color corresponds to one functional cluster, with cluster representatives highlighted in bold. See Supplementary Table S2 for detailed information. (**C**) Inhibition of the proteasome activity reduces APE1 truncation in OCI/AML3 cells. Representative Western blotting analysis on OCI/AML3 cells treated with 10 µM of MG132 and 100 nM of BFA for 4 h. Results showed a reduced protease activity in response to MG132. LC3-I to LC3-II conversion was used as positive control of autophagy induction. Actin was used as loading control. The amounts of the APE1 cleaved protein band, normalized to untreated cells, are indicated under each corresponding lane. (**D**) APE1 recombinant proteins (APE1^WT^ and APE1^K4pleA^) were incubated in the absence or in the presence of the 20S proteasome (20S PR); reaction products were analyzed by 12% SDS-PAGE and Western blotting. Asterisks indicate cleavage products. APE1^NΔ33^ mutant was loaded as a control for molecular mass comparison.

In order to identify the protease(s) responsible for APE1 proteolysis in the OCI/AML3 cell line, we first sub-fractionated the cell extracts to limit the proteome size to a specific subcellular compartment. We separately analyzed the cleavage activity of nuclear and cytoplasmic compartments on APE1^WT^ (Figure 4A, left). The efficacy of the biochemical procedure of sub-fractionation and isolation of the nuclear and cytoplasmic proteome was confirmed by the specific enrichment of the two compartment markers LSD1 (for the nucleus) and tubulin (for the cytoplasm). Testing the resulted extracts for proteolytic activity on APE1^WT^, only the cytoplasmic extract exhibited a specific activity that was inhibited by PMSF treatment, which is a specific inhibitor of serine-like proteases (Figure S2).

For the identification of the protease(s) involved in APE1 truncation, a chromatographic approach was combined with proteomic analysis. In particular, cytoplasmic extracts of OCI/AML3 cells were at first sub-fractionated through two subsequent chromatographic steps, a cationic-exchange-based process (on SP resin) followed by an affinity chromatography-based purification (on a benzamidine column for serine-like proteases) (Figure 4A, right). In both cases, the eluted fractions were then tested for protease activity using the recombinant full-length APE1 protein as substrate (Figure S2). This strategy allowed a recovery of the protease(s) of interest in the flow through of the SP column, and then in specific fractions of the benzamidine-based chromatography, when the above-mentioned flow through was loaded on the second purification step. The latter fractions were then subjected to further proteomic analysis for protein identification (Supplementary Table S1). In order to define whether particular proteolytic cascades may be involved in the degradation of the APE1 protein, we then performed functional enrichment analysis of the proteins here identified, using the Cytoscape plugin ClueGO (Figure 4B). Data analysis using the KEGG pathway database (http://www.genome.jp/kegg/pathway.html) highlighted an association with the autophagic flux. In particular, the CTSZ (Cathepsin Z) and CTSC (Cathepsin C) proteases are involved in the lysosomal pathway [26]. Remarkably, a significant enrichment (*p*-adj ≤ 0.05) in proteasomal components was identified in the positive fractions. In fact, PSMB1, PSMB3, PSMB4, PSMB5, PSMB6, PSMA3, PSMA4 and PSMA5 are some of the proteasome subunits associated with the proteasome pathway enrichment (see Table S2 for the complete list of proteins) [27].

In order to investigate the possible involvement of the proteasome in the observed APE1 cleavage, we treated OCI/AML3 cells with an inhibitor of the proteasomal activity. In particular, we treated OCI/AML3 cells with 10 µM MG132 for 4 h. To exclude any secondary effects due to MG132 treatment in a cell context, we performed *in vitro* proteolysis assay pretreating OCI/AML3 cell extracts with MG132 (Figure S3). As apparent in Figure 4C and in Figure S3, MG132 treatment highly reduced APE1 cleavage, confirming an involvement of the proteasome machinery in the above-mentioned reactions. Interestingly, the proteolytic degradation was not ascribable to the autophagic pathway, because the presence of the autophagy inhibitor bafilomycin A1 (BFA) did not hamper APE1 cleavage. To definitively demonstrate the involvement of the proteasome in APE1 truncation, we treated purified recombinant APE1 forms with purified 20S proteasome (Figure 4D). APE1^WT^ and APE1^K4pleA^ recombinant proteins were incubated with the catalytic subunit of the 20S proteasome (20S PR), and the cleavage activity was inspected by Western blotting analysis. As shown in Figure 4D, different cleavage products were observed in the case of APE1^WT^ and APE1^K4pleA^, further supporting a proteasomal-mediated cleavage of APE1.

## 4. Discussion

The pursuit of potential multi-pathway regulators in acute myeloid leukemia has led to the identification of APE1 as a potential therapeutic target [13,28]. Apart from its crucial role as a DNA-repair enzyme, APE1 is a multifunctional protein acting as a unique nuclear redox factor [29]. Indeed, several studies have shown that through the control of the DNA binding activity of different transcription factors (i.e., RAR, NF-ĸB, p53, AP-1 and STAT3) [30], APE1 regulates downstream transcriptional programs that are important in leukemia cell biology [31]. Therefore, the blockade of the APE1 redox function, e.g., with compound APX3330, has been extensively characterized and represents a promising strategy to alter cell survival and growth of different type of solid tumors and leukemias (acute lymphoblastic leukemia in particular) [31].

However, as knowledge of APE1 different functions has emerged, it has become clear that the development of APE1 redox and/or endonuclease inhibitors could not be the only choice for chemotherapeutical strategies. Recent evidence advocates for a better understanding of dysregulation of APE1 functions due to the impairment of its protein–protein interactions and PTMs. Although this represents a relatively neglected area, several studies have shown that different diseases are influenced by the dysregulation of DNA-repair enzyme protein networks [32]. Similarly, APE1 does not operate as an isolated protein, but rather dynamically interacts with a multitude of protein partners that coordinate and influence its activities [33].

The N-terminal portion of APE1 (35 amino acids) represents the region where the majority of these interactions [23] and PTMs [34] take place. Among PTMs, APE1 acetylation was highly characterized in terms of structural assignment and consequent biological outcomes. Specifically, acetylated residues (Lys27, Lys31, Lys32 and Lys35) are clustered in the protein N-terminus and are involved in protein–protein interactions [7], APE1 binding to RNAs and DNAs [35], endonuclease activity [5,15,35] and protein binding to nCaRE sequences [36]. Moreover, these residues are ubiquitinated by several ubiquitin ligases (e.g., MDM2, UBR3 and Parkin) [8,37,38], which thus control the steady state level of the protein. Additionally, APE1 enzymatic activities are controlled by interactions involving its N-terminal region [23]. In this context, Nucleophosmin 1 (NPM1) is the most studied APE1 partner, and represents a paradigmatic example of how dysregulation of this interaction could be envisioned as a therapeutic strategy [13,39]. Recently, we have characterized that NPM1 stimulates APE1 BER activity and, most importantly, AML patients bearing NPMc+ were more responsive to chemotherapy, due to the consequent APE1 accumulation into the cytoplasm [13]. This impaired relocalization of APE1 resulted in BER deficiencies and constituted a good prognostic factor for chemotherapy. Notably, we have demonstrated that there is an accumulation of the APE1 N-terminal truncated form in this cellular context. This proteolytic processing is a known APE1 modification; however, the exact impact of this process is not clear at present, and it is not known whether it is exclusive of certain cell types and/or cellular stress conditions. To date, the protease(s) involved in this process remain elusive, although a role was preliminary ascribed to granzyme A (GzmA) in T lymphocytes [11].

Accordingly, here we deepened the nature of APE1 cleavage in OCI/AML cells by identifying responsible proteases. First, the use of different APE1 N-terminal mutants and a synthetic peptide covering the 26–36 region allowed us to demonstrate that Lys31 and Lys32 are the major cleavage sites in the APE1 N-terminal region. In particular, the mutant APE1^K4pleA^, in which the four lysine residues Lys27, Lys31, Lys32 and Lys35 are mutated to alanine to mimic a constitutive acetylation, appeared to be associated with a decreased hydrolysis kinetics, compared to wild-type counterpart, thus suggesting a protective role of acetylation in APE1 proteolysis. Afterwards, we performed a dedicated proteomic analysis for the identification of proteases responsible for the cleavage in OCI/AML3 cells. To optimize the identification protocol and limit possible false positive results, we sub-fractioned cell extracts into a cytoplasmic compartment, where the major APE1-truncated form accumulates, and we performed two chromatography steps to enhance the recovery of putative proteases. Of note, collected fractions were tested for protease activity over recombinant APE1 before being subjected to proteomic analysis. Overall, a large number of proteins were identified in protease assay-positive fractions, which were further functionally characterized. Among pathways identified as having the highest probability, we focused on the proteasomal machinery as a potential candidate for further investigations.

Preliminary *in vitro* analysis preformed in OCI/AML3 cells with MG132, a known inhibitor of the proteasome, showed a reduced APE1 N-terminal cleavage, thus supporting proteomic results. As far as we know, this is the first time that the proteasome machinery has been linked to APE1 N-terminal proteolysis. In addition, exploratory experiments with purified proteins suggested a possible role of the 20S proteasome. Further analyses are undoubtedly needed to elucidate the proteasomal mechanism of this action. It is known that the 20S proteasome can partially cleave proteins that contain unfolded regions in an ubiquitin-independent process, leading to the formation of cleaved protein with altered functionality [40]. The fact that the N-terminal region of APE1 has been characterized as an intrinsically disordered region [41] reinforces the hypothesis that it can act as an accessible target for the 20S proteasome activity. Many issues regarding this possible regulation remain to be explored. Specifically, we need to clarify the contribution of APE1 PTMs and interactors involving the N-terminal region in protecting/stimulating proteasomal degradation. Furthermore, we need to elucidate if this protein truncation process depends on particular stimuli and/or pathological conditions, although some preliminary data suggested that serum starvation could prompt APE1 truncation (data not shown). Finally, further studies are needed to understand whether this N-terminal truncated form generates novel protein species with functional properties distinct from the intact protein. Expanding our knowledge in this area may pave the way toward a deeper understanding of the APE1 relevance in cancer biology. Though preliminary, our results represent an interesting opportunity for cancer medicine, as the use of proteasome inhibitors is now considered and encouraged in therapeutic interventions, especially in acute leukemia [40,42].

## Supplementary Materials

The following are available online at https://www.mdpi.com/2218-273X/10/4/531/s1, Figure S1: Time-course analysis of hydrolysis of internally-quenched fluorescent APE1 26-36 peptide at different concentrations; Figure S2: Protease activity tested on purified fractions from benzamidine-based chromatography; Figure S3: Proteasomal inhibitor MG132 decreases APE1 cleavage; Table S1: Identification details of identified proteins reported in this study; Table S2: Cytoscape/ClueGO functional annotation of the proteins in the positive fractions sent to mass spectrometry.

## References

[1] Sung J.S., Demple B. (2006). Roles of base excision repair subpathways in correcting oxidized abasic sites in DNA. Febs J..

[2] Mitra S., Izumi T., Boldogh I., Bhakat K., Chattopadhyay R., Szczesny B. (2007). Intracellular trafficking and regulation of mammalian AP-endonuclease 1 (APE1), an essential DNA repair protein. DNA Repair.

[3] Vascotto C., Fantini D., Romanello M., Cesaratto L., Deganuto M., Leonardi A., Radicella J.P., Kelley M.R., D’Ambrosio C., Scaloni A. (2009). APE1/Ref-1 Interacts with NPM1 within Nucleoli and Plays a Role in the rRNA Quality Control Process. Mol. Cell. Biol..

[4] Antoniali G., Serra F., Lirussi L., Tanaka M., D’Ambrosio C., Zhang S., Radovic S., Dalla E., Ciani Y., Scaloni A. (2017). Mammalian APE1 controls miRNA processing and its interactome is linked to cancer RNA metabolism. Nat. Commun..

[5] Fantini D., Vascotto C., Marasco D., D’Ambrosio C., Romanello M., Vitagliano L., Pedone C., Poletto M., Cesaratto L., Quadrifoglio F. (2010). Critical lysine residues within the overlooked N-terminal domain of human APE1 regulate its biological functions. Nucleic Acids Res..

[6] Bhakat K.K., Sengupta S., Adeniyi V.F., Roychoudhury S., Nath S., Bellot L.J., Feng D., Mantha A.K., Sinha M., Qiu S. (2016). Regulation of limited N-terminal proteolysis of APE1 in tumor via acetylation and its role in cell proliferation. Oncotarget.

[7] Lirussi L., Antoniali G., Vascotto C., D’Ambrosio C., Poletto M., Romanello M., Marasco D., Leone M., Quadrifoglio F., Bhakat K.K. (2012). Nucleolar accumulation of APE1 depends on charged lysine residues that undergo acetylation upon genotoxic stress and modulate its BER activity in cells. Mol. Biol. Cell.

[8] Busso C.S., Iwakuma T., Izumi T. (2009). Ubiquitination of mammalian AP endonuclease (APE1) regulated by the p53-MDM2 signaling pathway. Oncogene.

[9] Busso C.S., Wedgeworth C.M., Izumi T. (2011). Ubiquitination of human AP-endonuclease 1 (APE1) enhanced by T233E substitution and by CDK5. Nucleic Acids Res..

[10] Chattopadhyay R., Wiederhold L., Szczesny B., Boldogh I., Hazra T.K., Izumi T., Mitra S. (2006). Identification and characterization of mitochondrial abasic (AP)-endonuclease in mammalian cells. Nucleic Acids Res..

[11] Fan Z., Beresford P.J., Zhang D., Xu Z., Novina C.D., Yoshida A., Pommier Y., Lieberman J. (2003). Cleaving the oxidative repair protein Ape1 enhances cell death mediated by granzyme A. Nat. Immunol..

[12] Guo Y., Chen J., Zhao T., Fan Z. (2008). Granzyme K degrades the redox/DNA repair enzyme Ape1 to trigger oxidative stress of target cells leading to cytotoxicity. Mol. Immunol..

[13] Vascotto C., Lirussi L., Poletto M., Tiribelli M., Damiani D., Fabbro D., Damante G., Demple B., Colombo E., Tell G. (2014). Functional regulation of the apurinic/apyrimidinic endonuclease 1 by nucleophosmin: Impact on tumor biology. Oncogene.

[14] Antoniali G., Lirussi L., Poletto M., Tell G. (2014). Emerging roles of the nucleolus in regulating the DNA damage response: The noncanonical DNA repair enzyme APE1/Ref-1 as a paradigmatical example. Antioxid. Redox Signal..

[15] Burra S., Marasco D., Malfatti M.C., Antoniali G., Virgilio A., Esposito V., Demple B., Galeone A., Tell G. (2019). Human AP-endonuclease (Ape1) activity on telomeric G4 structures is modulated by acetylatable lysine residues in the N-terminal sequence. DNA Repair.

[16] Ciaccio C., Tundo G.R., Grasso G., Spoto G., Marasco D., Ruvo M., Gioia M., Rizzarelli E., Coletta M. (2009). Somatostatin: A novel substrate and a modulator of insulin-degrading enzyme activity. J. Mol. Biol..

[17] Hochegger K., Eller P., Huber J.M., Bernhard D., Mayer G., Zlabinger G.J., Rosenkranz A.R. (2007). Expression of granzyme A in human polymorphonuclear neutrophils. Immunology.

[18] D’Ambrosio C., Arena S., Fulcoli G., Scheinfeld M.H., Zhou D., D’Adamio L., Scaloni A. (2006). Hyperphosphorylation of JNK-interacting protein 1, a protein associated with Alzheimer disease. Mol. Cell Proteom..

[19] Bruschi M., Sinico R.A., Moroni G., Pratesi F., Migliorini P., Galetti M., Murtas C., Tincani A., Madaio M., Radice A. (2014). Glomerular autoimmune multicomponents of human lupus nephritis in vivo: α-enolase and annexin AI. J. Am. Soc. Nephrol..

[20] Shannon P., Markiel A., Ozier O., Baliga N.S., Wang J.T., Ramage D., Amin N., Schwikowski B., Ideker T. (2003). Cytoscape: A software environment for integrated models of biomolecular interaction networks. Genome Res..

[21] Bindea G., Mlecnik B., Hackl H., Charoentong P., Tosolini M., Kirilovsky A., Fridman W.-H., Pagès F., Trajanoski Z., Galon J. (2009). ClueGO: A Cytoscape plug-in to decipher functionally grouped gene ontology and pathway annotation networks. Bioinformatics.

[22] Yoshida A., Urasaki Y., Waltham M., Bergman A.-C., Pourquier P., Rothwell D.G., Inuzuka M., Weinstein J.N., Ueda T., Appella E. (2003). Human Apurinic/Apyrimidinic Endonuclease (Ape1) and Its N-terminal Truncated Form (AN34) Are Involved in DNA Fragmentation during Apoptosis. J. Biol. Chem..

[23] Vascotto C., Cesaratto L., Zeef L.A., Deganuto M., D’Ambrosio C., Scaloni A., Romanello M., Damante G., Taglialatela G., Delneri D. (2009). Genome-wide analysis and proteomic studies reveal APE1/Ref-1 multifunctional role in mammalian cells. Proteomics.

[24] Kummer J.A., Kamp A.M., Citarella F., Horrevoets A.J., Hack C.E. (1996). Expression of human recombinant granzyme A zymogen and its activation by the cysteine proteinase cathepsin C. J. Biol. Chem..

[25] Yoshida A., Pourquier P., Pommier Y. (1998). Purification and characterization of a Mg2+-dependent endonuclease (AN34) from etoposide-treated human leukemia HL-60 cells undergoing apoptosis. Cancer Res..

[26] Fu Z., Wang B., Wang S., Wu W., Wang Q., Chen Y., Kong S., Lu J., Tang Z., Ran H. (2014). Integral proteomic analysis of blastocysts reveals key molecular machinery governing embryonic diapause and reactivation for implantation in mice. Biol. Reprod..

[27] Moiseeva T.N., Bottrill A., Melino G., Barlev N.A. (2013). DNA damage-induced ubiquitylation of proteasome controls its proteolytic activity. Oncotarget.

[28] Ding J., Fishel M.L., Reed A.M., McAdams E., Czader M., Cardoso A.A., Kelley M.R. (2017). Ref-1/APE1 as Transcriptional Regulator and Novel Therapeutic Target in Pediatric T-cell Leukemia. Mol. Cancer.

[29] Kelley M.R., Georgiadis M.M., Fishel M.L. (2012). APE1/Ref-1 role in redox signaling: Translational applications of targeting the redox function of the DNA repair/redox protein APE1/Ref-1. Curr. Mol. Pharm..

[30] Fishel M.L., Colvin E.S., Luo M., Kelley M.R., Robertson K.A. (2010). Inhibition of the Redox Function of APE1/Ref-1 in Myeloid Leukemia Cell Lines Results in a Hypersensitive Response to Retinoic Acid-induced Differentiation and Apoptosis. Exp. Hematol..

[31] Shah F., Logsdon D., Messmann R.A., Fehrenbacher J.C., Fishel M.L., Kelley M.R. (2017). Exploiting the Ref-1-APE1 node in cancer signaling and other diseases: From bench to clinic. Npj Precis Oncol..

[32] Limpose K.L., Corbett A.H., Doetsch P.W. (2017). BERing the burden of damage: Pathway crosstalk and posttranslational modification of base excision repair proteins regulate DNA damage management. DNA Repair.

[33] Thakur S., Dhiman M., Tell G., Mantha A.K. (2015). A review on protein–protein interaction network of APE1/Ref-1 and its associated biological functions. Cell Biochem. Funct..

[34] Busso C.S., Lake M.W., Izumi T. (2010). Posttranslational modification of mammalian AP endonuclease (APE1). Cell Mol. Life Sci..

[35] Poletto M., Vascotto C., Scognamiglio P.L., Lirussi L., Marasco D., Tell G. (2013). Role of the unstructured N-terminal domain of the hAPE1 (human apurinic/apyrimidinic endonuclease 1) in the modulation of its interaction with nucleic acids and NPM1 (nucleophosmin). Biochem. J..

[36] Antoniali G., Lirussi L., D’Ambrosio C., Dal Piaz F., Vascotto C., Casarano E., Marasco D., Scaloni A., Fogolari F., Tell G. (2014). SIRT1 gene expression upon genotoxic damage is regulated by APE1 through nCaRE-promoter elements. Mol. Biol. Cell.

[37] Meisenberg C., Tait P.S., Dianova I.I., Wright K., Edelmann M.J., Ternette N., Tasaki T., Kessler B.M., Parsons J.L., Kwon Y.T. (2012). Ubiquitin ligase UBR3 regulates cellular levels of the essential DNA repair protein APE1 and is required for genome stability. Nucleic Acids Res..

[38] Scott T.L., Wicker C.A., Suganya R., Dhar B., Pittman T., Horbinski C., Izumi T. (2017). Polyubiquitination of apurinic/apyrimidinic endonuclease 1 by Parkin. Mol. Carcinog..

[39] Poletto M., Malfatti M.C., Dorjsuren D., Scognamiglio P.L., Marasco D., Vascotto C., Jadhav A., Maloney D.J., Wilson D.M., Simeonov A. (2015). Inhibitors of the apurinic/apyrimidinic endonuclease 1 (APE1)/nucleophosmin (NPM1) interaction that display anti-tumor properties. Mol. Carcinog..

[40] Kumar Deshmukh F., Yaffe D., Olshina M.A., Ben-Nissan G., Sharon M. (2019). The Contribution of the 20S Proteasome to Proteostasis. Biomolecules.

[41] Marasco D., Scognamiglio P.L. (2015). Identification of inhibitors of biological interactions involving intrinsically disordered proteins. Int. J. Mol. Sci..

[42] Cloos J., Roeten M.S., Franke N.E., van Meerloo J., Zweegman S., Kaspers G.J., Jansen G. (2017). (Immuno)proteasomes as therapeutic target in acute leukemia. Cancer Metastasis Rev..

