# Supplementary material for: Cleavage of the APE1 N-Terminal Domain in Acute Myeloid Leukemia Cells Is Associated with Proteasomal Activity
Source: Biomolecules. 2020 Mar 31;10(4):531. doi: 10.3390/biom10040531 (PMC7226146; doi:10.3390/biom10040531)
Supplement: Supplementary file 1 [file biomolecules-10-00531-s001.zip › biomolecules-747753 proofed supplementary/biomolecules-747753 proofed supplementary figures.pdf]

# Figure S1

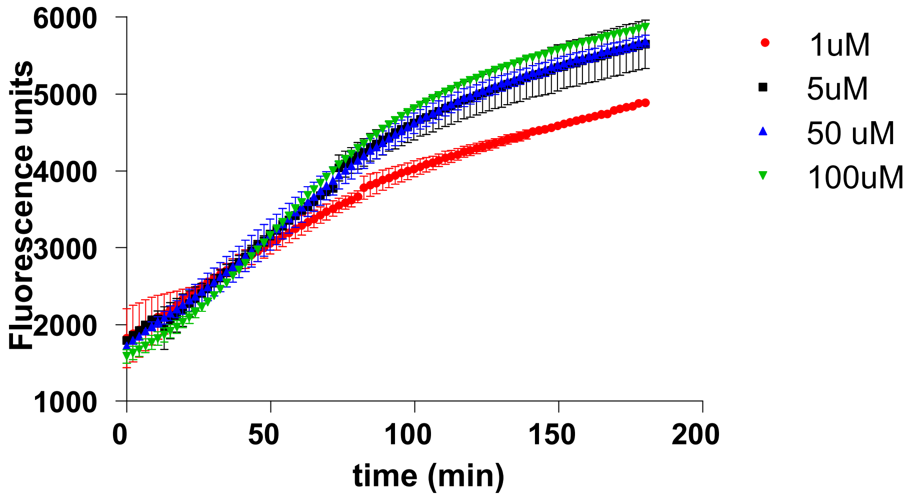


Figure S1. Time-course analysis of hydrolysis of internally-quenched fluorescent APE1 26-36 peptide at different concentrations. The peptide substrate was incubated with the cell lysate in low-volume 384-well plates at different concentrations, for 200 min. Fluorescence emitted at 460 nm was monitored upon excitation at 330 nm. A fluorescence signal is generated when the substrate is cleaved.

# Figure S2

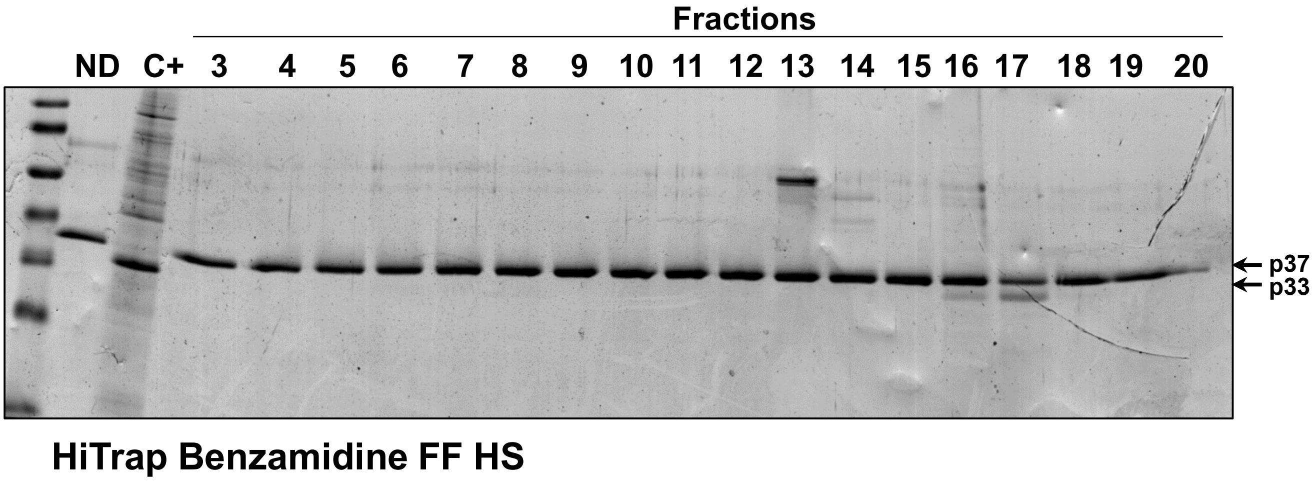


Figure S2. Protease activity tested on purified fractions from benzamidine-based chromatography. Representative Coomassie staining showing *in vitro* proteolysis for the fractions (3-20) eluted from the HiTrap Benzamidine on rAPE1 protein (300 ng), as described in Material and Methods section. The positive fractions (16 and 17) were pooled and analyzed through mass spectrometry for protein identification. APE1 full length (p37) and its truncated form (p33) are indicated on the right side of the figure.

# Figure S3

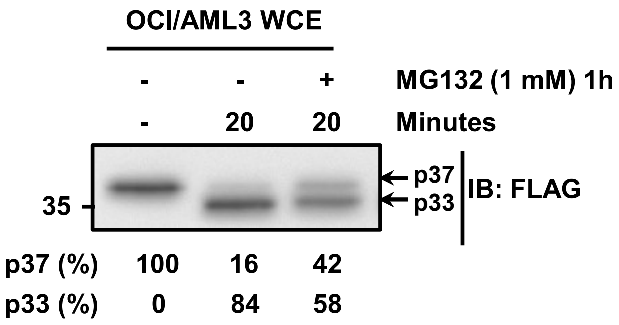


Figure S3. Proteosomal inhibitor MG132 decreases APE1 cleavage. Representative Western blotting analysis on *in vitro* proteolysis reactions where OCI/AML3 whole cell extracts (10 μg) were pre-treated with MG132 (1 mM) showing the slight inhibitory effect of MG132 in preventing APE1 truncation. Antibody specific for the FLAG-tag was used. Percentage of the conversion from APE1 full length (p37) to the truncated form (p33) are indicated below each corresponding lane.
